# Learning from cerebrospinal fluid drug-resistant HIV escape-associated encephalitis: a case report

**DOI:** 10.1186/s12985-023-02255-0

**Published:** 2023-12-10

**Authors:** Jing Kang, Ziqiu Wang, Ying Zhou, Wen Wang, Ying Wen

**Affiliations:** 1 National Health Commission (NHC) Key Laboratory of AIDS Immunology (China Medical University), National Clinical Research Center for Laboratory Medicine, The First Hospital of China Medical University, Shenyang, China; 2 Key Laboratory of AIDS Immunology, Chinese Academy of Medical Sciences, Shenyang, China; 3 Dongguan Institute for Microscale and Precision Medical Measurement, Dongguan, China; 4 Department of Infectious Diseases, The First Affiliated Hospital of China Medical University, No. 155, Nanjing North Street, Heping District, Shenyang, 110001 Liaoning Province China

**Keywords:** HIV, Antiretroviral therapy, CSF viral escape, Encephalitis, Drug resistance

## Abstract

**Background:**

In the era of antiretroviral therapy (ART), central nervous system (CNS) complications in patients with human immunodeficiency virus (HIV) infection are sometimes associated with cerebrospinal fluid (CSF) viral escape. Here, we reported a case of persistent CNS viral escape with recurrent symptomatic encephalitis, which had ultimate stabilization achieved by a combination of ART adjustment and corticosteroids.

**Case presentation:**

A 27-year-old man with HIV infection complained of recurrent headaches during the last year. His magnetic resonance imaging (MRI) presented diffused bilateral white matter lesions, and laboratory tests confirmed elevated CSF protein level, lymphocytic pleocytosis, and detectable CSF HIV RNA (774 copies/mL). Plasma HIV RNA was well suppressed with tenofovir, lamivudine, and lopinavir/ritonavir. Prednisone 60 mg once daily was initiated to reduce intracranial inflammation, followed by a good clinical response, with CSF HIV RNA still detectable (31.1 copies/mL). During the gradual tapering of prednisone, his headache relapsed, and booming viral loads were detected in both CSF (4580 copies/mL) and plasma (340 copies/mL) with consistent drug-resistant mutations. Thereupon, prednisone was resumed and the ART regimen was switched to zidovudine, lamivudine, and dolutegravir according to drug resistance tests. Persistent clinical recovery of symptoms, neuroimaging, and laboratory abnormalities were observed in the follow-up visits.

**Conclusion:**

CSF and plasma HIV RNA and further drug resistance tests should be monitored in HIV-infected patients with neurologic symptoms, as opportunistic infections or tumors can be ruled out. ART optimization using a sensitive regimen may be crucial for addressing CSF viral escape and the related encephalitis.

## Background

The central nervous system (CNS) complications among people with human immunodeficiency virus (HIV) infection could be caused by opportunistic infections, neoplastic lesions, and immune dysfunctions, which may occur during different stages including antiretroviral therapy (ART)-naive, undergoing ART, ART interruption, immune reconstitution inflammatory syndrome (IRIS), and cerebrospinal fluid (CSF) viral escape [1, 2]. Encephalitis among HIV-positive individuals may be presented as HIV encephalitis, CMV encephalitis, PML at severe immunosuppressed stage, or CD8 encephalitis, autoimmune encephalitis, acute disseminated encephalomyelitis (ADEM) at relative immune recovery stage. Using CSF laboratory detection of cytological components/antigen/antibody/gene sequencing, in combination with neuroimaging examination even brain biopsy, etiological diagnosis of encephalitis could be identified. CSF HIV escape, with increasing clinical attention for inducing neurosymptoms, was defined as having a higher level of HIV RNA in CSF than in plasma [3], and the prevalence among ART-treated patients ranged from 5 to 20% [4]. Here, we report a case of CSF HIV escape with recurrent subacute neurological symptoms and subsequent emergence of drug resistance, who experienced a complete clinical improvement after the combination therapy with corticosteroids and ART optimization.

## Case presentation

**Fig.1:**
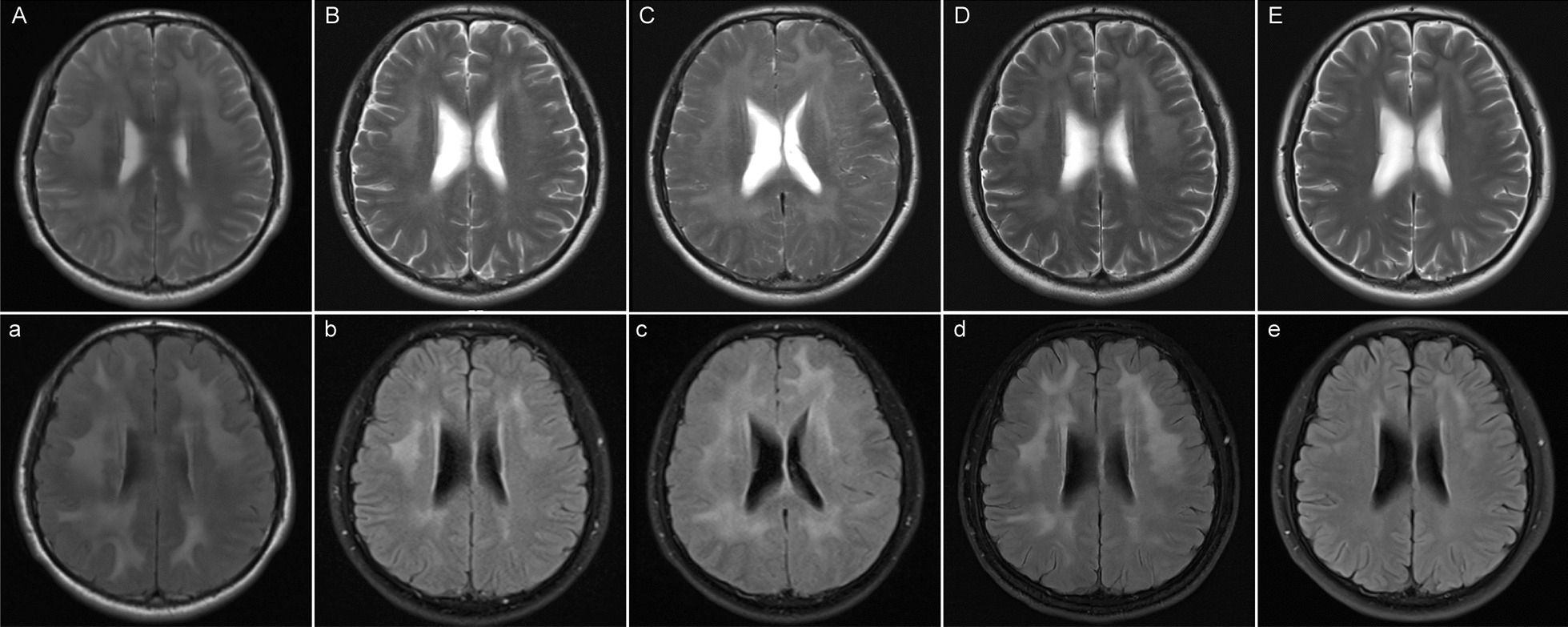
Cerebral MRI, laboratory examinations and treatment during each visit. T2-weighted (**A**) and T2-FLAIR images (**a**) on January 8th, 2022 showed diffused high signals of white matter under the bilateral frontoparietal temporal cortex, around the anterior and posterior corners of the lateral ventricles. T2-weighted (**B**) and T2-FLAIR images (**b**) on February 17th, 2022 presented obviously reduced area of multifocal leukoencephalopathy. T2-weighted (**C**) and T2-FLAIR images (**c**) on June 27th, 2022 showed significantly enlarged diffused white matter high signals on T2 and FLAIR sequence. T2-weighted (**D**) and T2-FLAIR images (**d**) on August 20th, 2022 showed slightly reduced affected brain area. T2-weighted (**E**) and T2-FLAIR images (**e**) on December 28th, 2022 presented remarkable improvement, leaving only slight white matter damage

A 27-year-old HIV-positive Chinese Manchu male complained of intermittent headache, nausea, and vomiting (non-projectile vomiting three times in total) for two weeks in January 2022. No fever, amaurosis, or unconsciousness were presented. He denied preceding travel or unusual animal exposures. He was diagnosed with HIV infection five years ago and nadir CD4^+^ T-cell count was 79 cells/μL. He initiated ART with tenofovir, lamivudine, and efavirenz, but treatment failure occurred one year later with plasma HIV RNA 2.19 × 10^5^ copies/mL and CD4^+^ T-cell count 14 cells/μL. The local doctor shifted the ART regimen to tenofovir, lamivudine, lopinavir/ritonavir without a genotypic drug resistance test, and his plasma HIV viral load was well controlled thereafter. Neurological physical examination showed no abnormalities. The brain magnetic resonance imaging (MRI) presented bilateral multiple white matter T2-weighted hyperintensities especially in the FLAIR sequence (Fig. 1A, a), without T1-wighted enhancement or mass effect. Lumbar puncture showed high intracranial pressure, lymphocytic pleocytosis, and elevated CSF protein level (Table 1). CSF HIV RNA was 774 copies/mL, while plasma HIV RNA was undetectable. The following blood tests all displayed negative results, including the T-cell enzyme-linked immuno-spot assay for tuberculosis, the syphilis rapid plasma reagin titer, *Toxoplasma gondii* IgG and IgM, cytomegalovirus (CMV) IgM, herpes simplex virus (HSV)1/2 IgM, Epstein-Barr virus (EBV)-IgM, galactomannan, 1,3-β-D-glucan, cryptococcus antigen, EBV-DNA, CMV-DNA. Besides, the CSF tests which detected acid-fast stain, India ink stain, cryptococcus antigen, culturing of bacteria and fungus, and CMV-DNA were also all negative. No other pathogens were confirmed by the next-generation sequencing in the CSF (using the MGISEQ-2000 platform in BGI PathoGenesis Pharmaceutical Technology, BGI-Shenzhen). The pathological phenotype of encephalitis was required, but the patient declined brain biopsy. CD8 encephalitis was suspected, and thus prednisone and glycerol fructose were prescribed empirically to reduce inflammation reactions in the brain.

**Table 1:** Laboratory examinations and treatment of corticosteroids and antiretroviral therapy (ART) during each visit

Time points	1 (January 8-19th, 2022)	2 (February 2th-March 1st, 2022)	3 (June 23th-July 1st, 2022)	4 (August 20th, 2022)	5 (December 12-30th, 2022)	Reference intervals
CSF HIV-RNA (copies/ml)	774	31.1	4580	undetectable	(-)	Lower detection limit: 20
Plasma HIV-RNA (copies/ml)	< 20	< 20	340	undetectable	(-)	Lower detection limit: 20
Resistance mutations in CSF RNA	(-)	(-)	Major PI-related mutations (V82F), NNRTI-related mutations (V106I and V179D), NRTI-related mutations (A62V, K65R, and M184V)	(-)	(-)	
Resistance mutations in plasma RNA	(-)	(-)	the same as that in CSF RNA	(-)	(-)	
CSF cell count (/L)	47 × 10^6^	18 × 10^6^	22 × 10^6^	12 × 10^6^	(-)	(0–8) × 10^6^
CSF protein (mg/L)	1250	903	1329	685	(-)	120–600
Lumbar puncture pressure (mmH_2_O)	260	200	162	360	(-)	80–180
CD4 + T cell count (cells/μL)	366	571	294	(-)	457	410–1590
CD8 + T cell count (cells/μL)	1565	1416	1184	(-)	1281	190–1140
Corticosteroid therapy	Prednisone orally (60 mg daily)	Tapering prednisone (60 mg daily followed by a reduction of 5 mg per week), withdrawal in May 2022	Prednisone orally (60 mg daily)	Tapering prednisone	Withdrawal	
ART regimen	TDF + 3TC + LPV/r	TDF + 3TC + LPV/r	TDF + 3TC + LPV/r	AZT + 3TC + DTG (modified in July 2022)	AZT + 3TC + DTG	

CSF, cerebrospinal fluid; PI, protease inhibitor; NNRTI, nonnucleoside reverse transcriptase inhibitor; NRTI, nucleoside reverse transcriptase inhibitor; TDF, tenofovir disoproxil fumarate;3TC, lamivudine; LPV/r, lopinavir/ritonavir; AZT, zidovudine; DTG, dolutegravir

**Fig.2:**
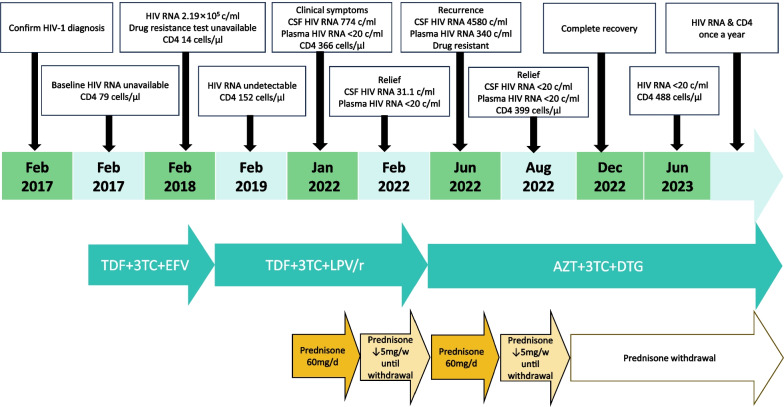
The timeline for treatment adjustment and follow up

One month later, the patient presented headache relief, and obvious improvement in MRI performance (Fig. 1B, b) and laboratory tests in the CSF (Table 1). Then, prednisone was tapered (60 mg daily followed by a reduction of 5 mg per week) and discontinued in May 2022. However, he complained of a headache relapse in June 2022. MRI showed the increased area of multifocal leukoencephalopathy (Fig. 1C, c). Moreover, increased HIV replication was observed in both CNS and plasma, and further HIV drug resistance tests of CSF and plasma (Sanger sequencing in Dongguan Medical Laboratory of Micro-scale and Presicion) presented identical results of major protease inhibitor (PI)-related mutations, nonnucleoside reverse transcriptase inhibitor (NNRTI)-related mutations, and nucleoside reverse transcriptase inhibitor (NRTI)-related mutations (Table 1). The encephalitis owning to CSF drug-resistant HIV escape was considered. Thus, ART regimen was switched to zidovudine (sensitive), lamivudine (highly resistant), and dolutegravir (sensitive). As well, prednisone 60 mg daily was restarted. One month after that, HIV suppression was achieved in both CSF and plasma (Table 1), and MRI displayed moderate shrink of diffused white matter area (Fig. 1D, d). Then, prednisone was tapered (the same as last time). A complete remission of brain MRI lesions was achieved in December 2022 (Fig. 1E, e). Meanwhile, prednisone had been withdrawn. The timeline for treatment adjustment and follow-up of this case was clearly outlined in Fig. 2.

## Discussion and conclusions

One characteristic of this case was the occurrence of subacute encephalitis caused by CSF drug-resistant HIV escape, which highlights the importance of CSF HIV RNA monitoring and the necessity of achieving sufficient CSF viral suppression under ART. The origin of CSF escape viral populations is either persistently replicating CNS reservoirs (likely in macrophages/microglia) or transient clonally expanded trafficking of infected T cells in the CNS [3]. Patients with CSF HIV escape may be symptomatic or asymptomatic [3, 5]. Active management was recommended for cases with symptomatic CSF HIV escape [6]. The neurosymptomatic CSF HIV escape with drug resistance mutations requires ART adjustment according to genotypic drug resistance results from both CSF and plasma, because the discordance of resistance patterns in different biologic compartments has been reported [7, 8].

The brain MRI of this case was characterized by bilateral and symmetrical FLAIR hyperintense abnormality throughout the cerebral white matter, which resembled previously reported CD8 encephalitis cases [9, 10]. Since the CD8 encephalitis case was first reported in 2004 [11] and given a name in 2013 [12], increasingly similar cases have been described. Although gadolinium contrast enhancing with the perivascular linear pattern was the typical imaging feature of CD8 encephalitis, some cases did not present MRI enhancement [13, 14]. Most CD8 encephalitis cases had a good response to corticosteroids, with which the survival rate was significantly higher than that of the patients with non-corticosteroid application [15]. For the cases that had no/transient response to corticosteroids, mycophenolate mofetil (MMF) might serve as a long-term steroid-sparing regimen [16]. It was a pity that the absence of brain biopsy and pathological proof for CD8 encephalitis was due to the refusal of the patient. A predominance of CD8^+^T-cell infiltration, while only weak HIV protein expression without giant multinuclear cells, is the characteristic histopathological finding among CD8 encephalitis cases [12]. The CSF characteristic presentation of this case was a marked lymphocytic pleocytosis, which may be associated with CSF HIV RNA replication, inflammation, and blood–brain barrier disruption. Although 90% (18/20) of CD8 encephalitis cases with subtyped CSF lymphocyte count demonstrated predominantly CD8^+^ T cells with inverted CD4/CD8 ratio, the CD4/CD8 ratio in CSF as a biomarker for CD8 encephalitis requires further investigation [17]. The transient response to corticosteroids in our case also suggested the underlying inflammatory injury mechanism. Importantly, a sustained response to ART optimization highlighted the role of targeting the underlying HIV replication, which is identical to a previous report [18]. Apart from classical HIV encephalitis or HIV leukoencephalopathy in ART-naive patients [19], we should pay attention to the encephalitis with symptomatic CNS HIV escape in ART-treated patients as a special clinical phenotype of HIV encephalitis.

In conclusion, HIV-positive patients with neurological complaints should be monitored for CSF HIV viral load and further genotypic resistance, if opportunistic infections or tumors can be excluded. Optimizing ART by using sensitive drugs may achieve a CSF viral suppression and a favorable outcome.

## Data Availability

All data from this study are included in this article.

## Abbreviations

CNS: Central nervous system
HIV: Human immunodeficiency virus
ART: Antiretroviral therapy
IRIS: Immune reconstitution inflammatory syndrome
CSF: Cerebrospinal fluid
ADEM: Acute disseminated encephalomyelitis
MRI: Magnetic resonance imaging
CMV: Cytomegalovirus
HSV: Herpes simplex virus
EBV: Epstein-Barr virus
PI: Protease inhibitor
NNRTI: Nonnucleoside reverse transcriptase inhibitor
NRTI: Nucleoside reverse transcriptase inhibitor
